# Applications and Predictors of Outcomes Following Stereo‐Electroencephalography in Pediatric Patients With Drug‐Resistant Epilepsy

**DOI:** 10.1111/cns.70332

**Published:** 2025-03-12

**Authors:** Qingzhu Liu, Chang Liu, Shuang Wang, Taoyun Ji, Yu Sun, Guojing Yu, Yao Wang, Hao Yu, Yuwu Jiang, Xiaoyan Liu, Lixin Cai

**Affiliations:** ^1^ Pediatric Epilepsy Center Peking University First Hospital Beijing China

**Keywords:** epilepsy prognosis, pediatric epilepsy, prognostic factors, stereo‐electroencephalography

## Abstract

**Aims:**

This study aims to evaluate the role of stereo‐electroencephalography (SEEG) in managing pediatric patients with drug‐resistant epilepsy. We further explore prognostic factors influencing surgical outcomes following SEEG‐guided resective or disconnective surgery.

**Methods:**

A retrospective review was conducted on pediatric patients who underwent SEEG at the Pediatric Epilepsy Center, Peking University First Hospital, between July 2017 and July 2022. Univariate and multivariate analyses identified key predictors for SEEG‐guided surgery. Kaplan–Meier survival analysis was employed to estimate the seizure‐free rate, and further statistical tests were applied to evaluate factors associated with seizure outcomes.

**Results:**

Among the 148 children included in this study, 102 underwent SEEG‐guided resective/disconnective surgery. Multivariate regression identified age at surgery (*p* < 0.05, 95% CI 0.190–0.997) as an independent predictor for selecting resective/disconnective surgery. The seizure‐free rate in patients who underwent SEEG‐guided surgery was 69.6%. Multivariate regression confirmed that total resection with lesional MRI (*p* < 0.05, 95% CI 0.012–0.186) and FCD type II (*p* < 0.05, 95% CI 0.051–0.851) were strong predictors of seizure freedom.

**Conclusions:**

SEEG plays a crucial role in pediatric epilepsy surgery, particularly in children under 6 years old. Total resection with lesional MRI and FCD type II was the most favorable prognostic predictor for achieving seizure freedom in children undergoing SEEG‐guided surgery.

## Graphical abstract

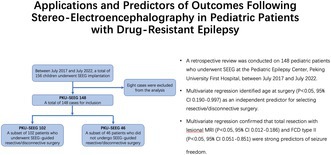

## 1 Introduction

For children with drug‐resistant epilepsy, resective or disconnective surgery remains one of the most effective interventions to control seizures and enhance quality of life [1, 2, 3]. In some cases, noninvasive preoperative evaluations can precisely localize the epileptogenic zone, facilitating the development of a targeted surgical plan [4]. However, in 25%–50% of cases, invasive intracranial electroencephalography (EEG) is necessary to delineate the epileptogenic zone for further evaluation [5].

Among invasive techniques, stereo‐electroencephalography (SEEG) has gained considerable traction in recent years, largely due to its safety profile, ability to explore multiple lobes without craniotomy, and its superior coverage of deeper brain regions [6]. The majority of pediatric SEEG studies have focused on the safety, precision, and efficacy of implantation [7, 8, 9, 10, 11], although these studies often involve relatively small cohorts [12, 13]. Additionally, few investigations have specifically addressed the differences in SEEG outcomes between children and adults [5, 14]. The aim of this study is to assess seizure outcomes and identify prognostic factors following SEEG in pediatric patients.

## 2 Materials and Methods

### 2.1 Inclusion and Exclusion Criteria

This retrospective study analyzed pediatric patients who underwent SEEG at the Pediatric Epilepsy Center of Peking University First Hospital between July 2017 and July 2022. This study was approved by the Peking University First Hospital ethics committee. All the participants' parents gave their written informed consent regarding the use of their children's data for research. The inclusion criteria were as follows: (1) age < 18 years at the time of surgery, (2) diagnosis of drug‐resistant epilepsy, with seizures remaining uncontrolled despite the standardized use of two or more anti‐seizure medications, and (3) unclear or inconsistent localization of the epileptogenic zone based on noninvasive preoperative evaluations, necessitating SEEG implantation for further clarification. Exclusion criteria included patients requiring radiofrequency thermocoagulation (RF‐TC) due to a diagnosis of hypothalamic hamartoma, as well as those with incomplete clinical data or those lost to follow‐up.

### 2.2 Pre‐Surgical Evaluation and SEEG Procedures

Pre‐surgical evaluations included a comprehensive clinical history, neurological examination, interictal and ictal video EEG, 3.0 T MRI, and PET/CT scans. SEEG was indicated following a multidisciplinary team discussion if the epileptogenic zone could not be localized with non‐invasive methods. The primary objectives of SEEG were as follows: (1) precise delineation of the epileptogenic zone to facilitate minimal cortical resection, (2) determination of the maximum extent of the epileptogenic zone for optimal cortical resection or disconnection, and (3) evaluation of limited epileptogenic zones correlated with eloquent cortex to assess the possibility of RF‐TC.

Electrode implantation was individualized based on non‐invasive findings and anatomo‐electro‐clinical correlations. Brain 3D MRI (3.0 T, 1 mm slice thickness, no gap) and MR angiography were performed under frameless and markerless conditions days or weeks before implantation. On the day of surgery, CT scans (1 mm slice thickness, no gap) were obtained with markers. All imaging data were integrated into the Sinovation surgical navigation software (Beijing, China) to design the electrode implantation plan. Electrodes (HuaKe HengSheng, Beijing, China), with a diameter of 0.8 mm, were implanted using the Sinovation surgical robot (Beijing, China) through oblique or orthogonal approaches. Each electrode had 8–16 contacts, with individual contacts measuring 2 mm in length and spaced 1.5 mm apart.

Post‐implantation CT scans (1 mm slice thickness, no gap) were performed within 24 h of the procedure. Intracranial EEG monitoring was conducted using a 128 or 256‐channel EEG system (NIHON KOHDEN EEG‐1200C, Japan). EEG signals were sampled at 2000 Hz and filtered between 5.3 and 600 Hz, with sensitivities set between 30 and 50 μV/cm. Habitual seizures were monitored, and electrical stimulation was applied to facilitate functional mapping and the localization of the epileptogenic zone. Electrical stimulation parameters included biphasic pulses (duration: 0.2 ms) delivered at 50 Hz for 5 s. The stimulation commenced at an intensity of 0.5 mA, incrementing by 0.5–2.0 mA until one of the following criteria was met: (1) a current of 6 mA, (2) a functional change, or (3) the occurrence of after‐discharges (ADs).

Based on SEEG findings and non‐invasive evaluation results, a final surgical decision was made. Children with a clearly defined epileptogenic zone who underwent resective or disconnective surgery were classified as the SEEG‐guided resective/disconnective surgery group, while those receiving other treatments were classified as the palliative group. When indicated, RFTC was performed before electrode removal, with power settings of 6–7 W sustained for 30 s.

### 2.3 Definitions of SEEG Onset Patterns and Prognosis

Ictal SEEG patterns were categorized into six types: localized seizure onset (less than one lobe), entire lobe, multilobar, multifocal onset, no seizures recorded, and clinical onset prior to EEG onset. The lobes analyzed included the frontal, temporal, parietal, occipital, and insular lobes. The distinction between multilobar and multifocal onset is as follows: a multilobar onset refers to a single seizure onset involving two or more adjacent lobes, whereas a multifocal onset involves at least two distinct seizure onsets occurring in either adjacent or remote lobes. Seizure outcomes were evaluated using the Engel classification system, with outcomes dichotomized as either good (Engel class I) or poor (Engel classes II–IV).

### 2.4 Statistical Analysis

All statistical analyses were performed using SPSS version 26.0 (IBM Corp., Armonk, NY, USA). All data should be subjected to tests for normality. Univariate analysis of seizure outcome predictors was conducted using chi‐squared tests for categorical variables. Variables with a *p* value of < 0.05 in the univariate analysis were subsequently entered into a multivariate logistic regression model using a backward elimination method. To analyze the effects of sex, sex was also entered into a multivariate logistic regression model. Kaplan–Meier survival curves were generated, and a Log‐Rank test was employed to compare seizure outcomes during a six‐year follow‐up period between the SEEG‐guided resective/disconnective surgery group and the non‐SEEG‐guided group. Additionally, linear regression analysis was conducted to examine temporal changes. A *p* value of < 0.05 was considered statistically significant in all analyses.

## 3 Results

Between July 2017 and July 2022, a total of 156 children underwent SEEG implantation at the Pediatric Epilepsy Center of Peking University First Hospital. Eight cases were excluded from the analysis due to hypothalamic hamartoma (*n* = 6), incomplete clinical data (*n* = 1), and loss to follow‐up (*n* = 1), leaving a total of 148 cases for inclusion in the study. Different subsets of patients were examined based on specific study aims:

- PKU‐SEEG148: All 148 patients who underwent SEEG implantation between July 2017 and July 2022.
- PKU‐SEEG102: A subset of 102 patients who underwent SEEG‐guided resective/disconnective surgery.
- PKU‐SEEG46: A subset of 46 patients who did not undergo SEEG‐guided resective/disconnective surgery.
- PKU‐SURG628: A population of 628 patients who underwent resective/disconnective surgery without SEEG guidance during the same period.

### 3.1 PKU‐SEEG148

**FIGURE 1:**
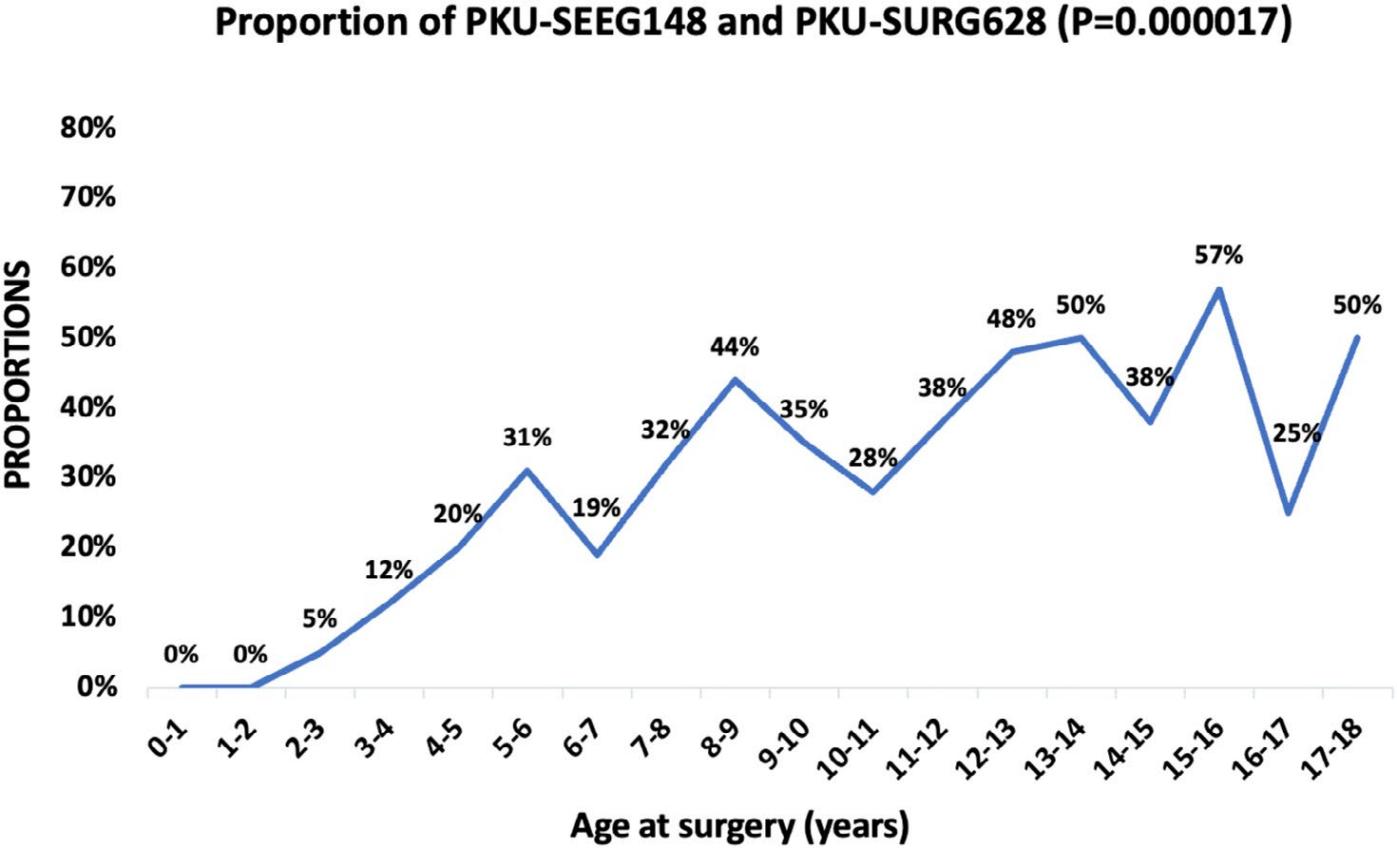
Proportion of PKU‐SEEG148 and PKU‐SURG628 by the age at surgery (blue line). Horizontal axis represented age at surgery. Vertical axis represented proportions of the number of SEEG patients (PKU‐SEEG148) and the number of patients undergoing directly resective/disconnective surgery (PKU‐SURG628) within the same age group. In our pediatric epilepsy center, the proportion of children following SEEG was significantly increased by the age at surgery (*p* = 0.000017).

This subset comprised 90 males and 58 females, with a median age at surgery of 8.0 years (range: 2.4–17.1 years). Of these, 51 patients (34.5%) were younger than 6 years. Four patients required two SEEG procedures, with a total of 1481 electrodes implanted. The median number of electrodes per procedure was 10.0 (range: 6–19). A unilateral approach was used in 121 patients (81.8%), while 27 patients underwent asymmetric bilateral implantation. The proportion of PKU‐SEEG148 to PKU‐SURG628 varied across age groups, showing a statistically significant increase with increasing age (Figure 1).

Following SEEG implantation, 102 patients underwent resective/disconnective surgery. Univariate analysis revealed that age at surgery below 6 years, positive MRI findings, and SEEG‐localized seizure onset pattern were significantly associated with the choice of resective/disconnective surgery (*p* < 0.05; Table 1). Multivariate regression confirmed that age at surgery under 6 years (*p* < 0.05; 95% CI 0.190–0.997) was an independent predictor of selecting resective/disconnective surgery (Table 2). The median follow‐up period for PKU‐SEEG148 was over 1 year, with 95 patients (64.2%) classified as Engel class I at the last follow‐up.

**TABLE 1:** Predictors of SEEG‐guided resective/disconnective surgery on univariate analysis.

Clinical characteristics	PKU‐SEEG102, *N* = 102	PKU‐SEEG46, *N* = 46	*p*
Sex, male	66	24	0.148
Age at surgery, under 6 years	41	10	0.029
Aura	16	5	0.437
Family history	9	5	0.764
Etiology, malformation of cortical development	90	39	0.561
Semiology, focal seizures	95	45	0.436
Clearly lateralized interictal EEG	50	25	0.548
Clearly lateralized ictal EEG	80	41	0.119
Concordant interictal and ictal EEG	71	28	0.296
Positive MRI	84	31	0.043
Abnormality on PET	96	40	0.192
Concordant ictal EEG and positive MRI	63	30	0.687
Concordant of MRI and PET abnormality	53	21	0.477
Unilateral hemisphere implant	81	40	0.271
Dominant hemisphere implant	54	18	0.120
SEEG ictal onset, localized seizure onset	77	27	0.039
Stimulation induced seizures	44	21	0.075

**TABLE 2:** Predictors of SEEG‐guided resective/disconnective surgery in multivariate analysis.

Factors	Odds ratio	95% confidence interval	*p*
Sex, male	2.082	0.966–4.487	0.061
Age at surgery, under 6 years	0.435	0.190–0.997	0.049
Positive MRI	2.286	0.971–5.382	0.058
SEEG ictal onset, localized seizure onset	0.454	0.206–1.002	0.051

In terms of complications, no serious or permanent morbidities were observed. Three patients developed intracranial hemorrhages, representing a complication rate of 0.2% per electrode (3/1481). No cerebrospinal fluid leaks or infections were reported.

### 3.2 PKU‐SEEG102

A total of 102 patients underwent SEEG‐guided resective/disconnective surgery; 22 patients had temporal lobe epilepsy, four patients had temporo‐parieto‐occipital epilepsy, and 76 patients had extratemporal lobe epilepsy. A total of 85 patients underwent tailored resection, 15 patients underwent temporal resection, and 70 patients underwent extratemporal resection. A total of 17 patients underwent disconnective surgery; seven patients underwent temporal disconnection, four patients underwent temporo‐parieto‐occipital disconnection, three patients underwent frontal disconnection, and three patients underwent other types of disconnection.

**FIGURE 2:**
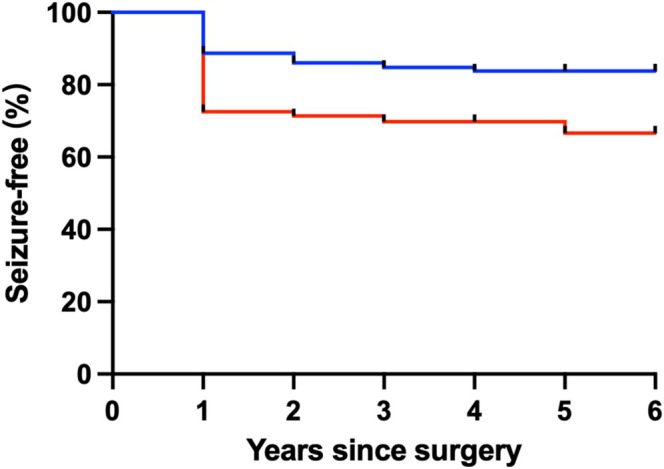
Kaplan–Meier analysis of time to seizure recurrence. Blue line represented PKU‐SURG628. Red line represented PKU‐SEEG102. Cumulative probability of continuous seizure freedom when comparing all patients, PKU‐SURG628 and PKU‐SEEG102. Of note, most relapses occurred in the first post‐surgical year.

Among the 102 patients in this subset, 71 (69.6%) achieved Engel class I at the last follow‐up. A significant difference in seizure‐free outcomes was observed between the PKU‐SEEG102 group and the PKU‐SURG628 group, with seizure‐free rates of 69.6% and 85.2%, respectively (Figure 2).

Univariate analysis (Table 3) identified several factors associated with favorable seizure outcomes: lateralized interictal EEG, positive MRI findings, PET metabolic abnormalities, concordant MRI and PET abnormalities, SEEG localized seizure onset pattern, total resection with lesional MRI, and focal cortical dysplasia (FCD) type II. Multivariate regression analysis further revealed that total resection with lesional MRI (*p* < 0.05; 95% CI 0.012–0.186) and post‐surgical pathology of FCD type II (*p* < 0.05; 95% CI 0.051–0.851) were independent predictors of favorable seizure outcomes (Table 4).

**TABLE 3:** Predictors of seizure outcomes in univariate analysis.

Clinical characteristics	Good outcome, *N* = 71	Poor outcome, *N* = 31	*p*
Sex, male	45	21	0.672
Age at surgery, under 6 years	28	13	0.813
Aura	12	4	0.771
Family history	6	3	1.000
Etiology, malformation of cortical development	61	29	0.337
Semiology, focal seizures	66	29	1.000
Clearly lateralized interictal EEG	41	9	0.008
Clearly lateralized ictal EEG	59	21	0.083
Concordant interictal and ictal EEG	51	20	0.460
Positive MRI	62	22	0.046
Abnormality on PET	70	26	0.010
Concordant ictal EEG and lesional MRI	48	15	0.066
Concordant of MRI and PET abnormality	44	9	0.002
Unilateral hemisphere implant	59	22	0.163
Dominant hemisphere implant	32	16	0.543
SEEG ictal onset, localized seizure onset	58	17	0.001
Stimulation induced seizures	43	15	0.253
Surgery involved insular lobe	25	13	0.518
Surgery involved rolandic area	23	11	0.761
Surgical procedure, resection	58	27	0.500
Total resection with lesional MRI	66	12	0.000
Focal cortical dysplasia type II	45	8	0.000

**TABLE 4:** Predictors of seizure outcomes in multivariate analysis.

Factors	Odds ratio	95% confidence interval	*p*
Sex	0.831	0.217–3.186	0.788
Clearly lateralized interictal EEG	0.669	0.197–2.272	0.519
Positive MRI	0.568	0.121–2.657	0.472
Abnormality on PET	0.531	0.040–6.976	0.630
Concordant of MRI and PET abnormality	1.369	0.265–7.075	0.708
SEEG ictal onset, localized seizure onset	3.509	0.873–14.103	0.077
Total resection with lesional MRI	0.047	0.012–0.186	0.000
Focal cortical dysplasia type II	0.208	0.051–0.851	0.029

### 3.3 PKU‐SEEG46

In the subset of 46 patients who did not undergo SEEG‐guided resective/disconnective surgery, 24 (52.5%) achieved Engel class I at the last follow‐up, following SEEG‐guided RFTC. The reasons for RFTC includeda focal epileptogenic zone (18 cases), 14 achieved seizure freedom after RFTC; close proximity of the epileptogenic zone to functional cortical areas (18 cases); and difficulty in localizing the epileptogenic zone via SEEG (10 cases). SEEG‐guided RFTC was performed based on interictal and ictal epileptic discharges.

## 4 Discussion

### 4.1 PKU‐SEEG148

SEEG, originally developed in France [15], has since gained widespread adoption across various global centers [16, 17]. This study, one of the largest pediatric SEEG reports in China, provides a summary of key characteristics related to pediatric SEEG cases. The proportion of PKU‐SEEG148 to PKU‐SURG628 across different age groups shows an increasing trend with age at surgery. The reasons for this are as follows:

First, in young children with drug‐resistant epilepsy, cortical malformations—the primary etiology—often involve multiple lobes or even an entire hemisphere [18]. Other common etiologies include Rasmussen encephalitis, Sturge–Weber syndrome, and long‐term epilepsy‐associated tumors (LEAT). In such cases, resective or disconnective surgeries are typically preferred without SEEG due to the extensive involvement. However, focal cortical dysplasia (FCD) is more frequently observed in children [19], where SEEG is often necessary to accurately localize the epileptogenic zone.

Second, epileptic spasms, which have limited localization value for identifying the epileptogenic zone, are more common in younger children. Even with SEEG, localizing the epileptogenic zone in these cases remains challenging due to the complexity of the epileptic spasm network [20]. In contrast, focal onset seizures are more common in adolescents, making SEEG more suitable for identifying anatomo‐electro‐clinical correlations.

Third, in cases of rolandic epilepsy, the strong plasticity of motor function in young children favors resective or disconnective surgeries under intraoperative motor evoked potential monitoring [21, 22]. Even if motor function is affected to some extent, rehabilitation can often achieve functional compensation. However, for adolescents with rolandic epilepsy, SEEG is preferred to precisely localize the epileptogenic zone and its relation to functional areas.

Lastly, with the aid of Sinovision software, the precise boundaries of structural lesions in young children can be delineated using 3D brain reconstructions [23], further supporting the preference for resective or disconnective surgeries.

Multivariate regression analysis indicates that SEEG‐guided resective or disconnective surgery is more common in children under 6 years old. On the one hand, young children tend to undergo this surgery if the epileptogenic zone affects motor function, as their functional plasticity allows for better recovery. On the other hand, young children often present with positive MRI and PET findings, increasing the likelihood of SEEG successfully localizing the epileptogenic zone.

### 4.2 PKU‐SEEG102

Unsurprisingly, seizure outcomes are better in children who undergo resective or disconnective surgery without SEEG recordings (Figure 2). This finding aligns with the results of Cardinale's study [24], which highlights the challenges of SEEG in more complex cases, especially those with negative MRI findings. Cardinale reported a 59.4% seizure‐free rate after a minimum two‐year follow‐up in a series of 470 cases [24], while Sivaraju reported a 60% seizure‐free rate in a series of 117 cases [25]. Compared to these studies on adult intracranial EEG, our study yielded better seizure outcomes in children. This can be attributed to several factors:

First, children undergoing resective or disconnective surgery without SEEG tend to have better outcomes compared to adults. Second, children with SEEG implantation may present with a higher proportion of positive MRI findings than adults. Furthermore, some children opt for multilobar resective or disconnective surgeries after SEEG implantation. Additionally, young children have greater functional plasticity than adults, making total resection or disconnection easier to achieve in this population.

Multivariate regression analysis found that total resection with lesional MRI findings and FCD type II are significantly associated with seizure freedom. These findings align with previous studies on seizure outcome predictors [24]. For children with positive MRI lesions, precise delineation of lesion boundaries using 3D brain reconstructions is crucial to minimize the risk of seizure recurrence. FCD type II often presents with distinct features in both MRI and interictal EEG patterns [26, 27, 28], including increased cortical thickness, the transmantle sign, gray‐white matter junction blurring, and gray matter signal changes [29]. Therefore, accurately identifying these features via MRI and 3D brain reconstruction is key to achieving seizure‐free outcomes following surgical resection.

### 4.3 PKU‐SEEG46

In the PKU‐SEEG46 group, none of the patients underwent resective or disconnective surgery; instead, SEEG‐guided RFTC was performed. Among the 18 cases with focal epileptogenic zones, 14 achieved seizure freedom after RFTC. Bourdillon reported only a 7% Engel class I outcome after a minimum 12‐month follow‐up [30]. The higher seizure‐free rate in our study can be explained by the fact that the epileptogenic zones in these cases were focal. Therefore, when designing the electrode implantation, it was essential to cover the epileptogenic zone comprehensively to maximize the effectiveness of RFTC. If the epileptogenic zone involves motor function, incomplete lesion removal during RFTC can increase the risk of seizure recurrence.

SEEG is a safe technique in pediatric populations [31, 32, 33]. The most serious complication associated with SEEG is intracranial hemorrhage, which was rare in our series due to the use of MR angiography and a surgical robot (Sinovation surgical robot, Beijing, China) that provided high accuracy. No cerebrospinal fluid leakage or infection occurred in our series. In young children, skull thickness is a key consideration, with a minimum thickness of 2.5 mm required to minimize the risk of cerebrospinal fluid leakage by ensuring the hollow screw reaches the inner plate of the bone.

### 4.4 Limitations of the Study

The primary limitation of this study is its retrospective design and single‐center scope. Additionally, we did not provide a detailed comparison of quality of life and neurological function in children before and after surgery. Since our center specializes in pediatric epilepsy, we did not include adult cases, preventing us from highlighting differences between pediatric and adult SEEG outcomes.

### 4.5 Conclusion

In conclusion, SEEG‐guided resective or disconnective surgery should be the first consideration to achieve seizure freedom and improve quality of life in pediatric epilepsy. Our study indicates that younger children (under 6 years of age) are more likely to undergo SEEG‐guided surgery, with total resection with lesional MRI findings and FCD type II being strong prognostic factors for favorable outcomes.

## Author Contributions

Lixin Cai, Yuwu Jiang, and Xiaoyan Liu designed this study and revised the manuscript. Chang Liu and Qingzhu Liu analyzed the data and drafted and revised the manuscript. Guojing Yu collected the data. Yu Sun, Hao Yu, and Yao Wang helped to select the patients. Taoyun Ji and Shuang Wang helped to interpret the EEG data. Guojing Yu followed the patients. All the authors contributed to the article and approved the submitted version.

## Conflicts of Interest

The authors declare no conflicts of interest.

## Data Availability

The data that support the findings of this study are available on request from the corresponding author. The data are not publicly available due to privacy or ethical restrictions.
